# Validation of brief screening instruments for internalizing and externalizing disorders in Mozambican adolescents

**DOI:** 10.1186/s12888-022-04189-3

**Published:** 2022-08-12

**Authors:** Kathryn L. Lovero, Salma Ebrahim Adam, Carolina Ezequias Bila, Elda D. Canda, Maria Eduarda Fernandes, Teresa I. Baltazar Rodrigues, Mariel C. Tai Sander, Claude A. Mellins, Cristiane S. Duarte, Palmira Fortunato dos Santos, Milton L. Wainberg

**Affiliations:** 1 Department of Sociomedical Sciences, Columbia University Mailman School of Public Health, 722 W. 168th St. 9th Floor, New York, NY 10032 USA; 2 Department of Mental Health, Ministry of Health, Maputo, Mozambique; 3 Department of Psychiatry, New York State Psychiatric Institute and Columbia University Vagelos College of Physicians and Surgeons, New York, NY USA; 4 HIV Center for Clinical and Behavioral Health, Department of Psychiatry, New York State Psychiatric Institute and Columbia University, New York, NY USA

**Keywords:** Youth, LMIC, Psychometrics, PHQ, GAD, SDQ, Depression, Anxiety, Disruptive behavior disorders, ADHD

## Abstract

**Background:**

Mental disorders are the leading cause of disability for youth worldwide. However, there is a dearth of validated, brief instruments to assess mental health in low- and middle-income countries (LMIC). We aimed to facilitate identification of mental disorders in LMIC contexts by adapting and validating measures of internalizing and externalizing disorders for adolescents in Mozambique, an LMIC in southeastern Africa.

**Methods:**

We selected instruments with good support for validity in high-income and other LMIC settings: the Patient Health Questionnaire Adolescent (PHQ-A), Generalized Anxiety Disorders 7 (GAD-7), and Strengths and Difficulties Questionnaire (SDQ). Instruments were adapted by local and international mental health specialists followed by cognitive interviews (*n* = 48) with Mozambican adolescents. We administered the instruments along with the Miniature International Neuropsychiatric Interview for Children and Adolescents (MINI-KID)to 485 adolescents aged 12–19 years attending two secondary schools in Maputo City, Mozambique. One week later, we re-administered instruments to a randomly selected sample of 49 adolescents.

**Results:**

Participants were 66.2% (*n* = 321) female and the average age was 15.9 (S.D = 1.7).Internal consistency (alpha = 0.80, PHQ-A; 0.84, GAD-7; 0.80, SDQ) and test–retest reliabilty (ICC = 0.74, PHQ-A; 0.70, GAD-7; 0.77, SDQ) were acceptabe for the PHQ-A, GAD-7, and the full SDQ. The SDQ internalizing subscale showed poor test–retest reliability (ICC = 0.63) and the SDQ externalizing subscale showed poor internal consistency (alpha = 0.65). All instruments demonstrated good sensitivity and specificity (> 0.70). Youden’s index identified optimal cutoff scores of 8 for the PHQ-A, 5 for the GAD-7, 10 for the SDQ internalizing and 9 for the SDQ externalizing subscales, though a range of scores provided acceptable sensitivity and specificity.

**Conclusions:**

Our data supports reliability and validity of the PHQ-A, GAD-7, and SDQ instruments for rapidly assessing mental health problems in Mozambican adolescents. Use of these tools in other contexts with limited specialists may asist with expanding mental health assessment. Specific instrument and cutoff selection should be based on screening goals, treatment resources, and program objectives.

**Supplementary Information:**

The online version contains supplementary material available at 10.1186/s12888-022-04189-3.

## Background

Globally, mental disorders are the largest contributor to burden of disease in adolescents, accounting for 45% of years lived with disability [1]. It is estimated that 90% of adolescents live in low-and middle-income countries (LMIC) [2] and that 10–20% of these adolescents have one or more mental disorders [3]. Around 75% of mental disorders have their onset prior to or during adolescence [4], and mental illness in youth is associated with poor lifelong health, educational, social and economic outcomes [5–14]. Thus, there is an urgent need to increase early identification and treatment of adolescents with mental disorders, particularly those in LMIC settings.

Despite the high estimated burden of mental disorders in LMIC adolescents, there is an enormous shortage of mental health providers in these regions and almost no specialized child and adolescent mental health services [15]. Task-shifting of mental health services and integration of mental health care into primary and community care settings have been proposed as solutions to the vast treatment gap in LMIC [16]. However, in the absence of an adequate cadre of trained mental health specialists, locally validated brief assessment instruments are needed to help accurately a) estimate the prevalence of mental disorders and efficiently target resources, b) screen for these disorders in primary and community care settings and triage for treatment, and c) evaluate the impact of task-shifted mental health interventions. A recent systematic review found just 17 studies that validated mental health screening tools in LMIC adolescents [17], only four of which were in sub-Saharan Africa, where nearly 20% of the world’s adolescents reside [18].

In Mozambique, a low-income country located in southeastern Africa, adolescents account for one-third of the country’s over 31 million inhabitants and are the fastest growing population group [19]. While mental health services are offered free-of-charge through the national health system, there are less than 500 specialists (psychiatrists, psychologists, and psychiatric technicians) nationwide [20]. The Mozambique Ministry of Health has included child and adolescent mental health as a priority in its Strategy and Action Plan for Mental Health 2016–2026 [21] and is working to implement task-shifted adolescent mental health services. However, like in other LMIC [22], there has been almost no research on adolescent mental health locally and, though two studies have demostrated the validity of brief mental health assessment tools adapted for Mozambican adults [23, 24], to our knowledge, none have been evaluated in Mozambican adolescents.

This study aimed to adapt commonly used brief screening tools to assess adolescent mental health problems and evaluate their measurment properties in Mozambican adolescents. We focused specifically on instruments previously validated in other LMIC adolescent populations to screen for internalizing disoders (depression and anxiety), as they are estimated to be the highest burden disorders in adolescents globally as well as in Mozambique [25], and externalizing disorders (conduct disorder, oppositional defiant disorder, and attention deficit hyperactivity disorder [ADHD]), as they are the strongly associated with poor educational and social outcomes [26]. Following a review of the literature and discussion with a team of international mental health experts, we selected the Patient Health Questionnaire Adolescent (PHQ-A) and the Generalized Anxiety Disorder 7 (GAD-7) because of their demonstrated performance identifying depression and anxiety, respectively, in adolescents of high-income countries and LMIC [27–32]. We chose to also adapt and evaluate the Strengths and Difficulties Questionnaire (SDQ), which assesses symptoms of both internalizing disorders and externalizing disorders, as it is one of the most widely used brief screening instruments in youth worldwide and the only open-access brief instrument for externalizing disorders, although it is not designed to discern specific disorders.

## Methods

### Sample

This cross-sectional study was conducted at two secondary schools in peripheral and central regions of the Mozambican capital, Maputo City. We selected these schools as typical of adolescents from lower and higher socioeconomic urban areas, respectively. During the school year our study was conducted, the peripheral urban school had enrolled a total of 3243 students (58.2% female); the central urban school had enrolled 2987 students (55.1% female).

The study population included adolescents aged 12–19 years in grades 8–12. We aimed to include 50 adolescents per grade at each school (*n* = 5 00 total participants) and randomly selected 2–3 classes (~ 100 students) per grade per school to be invited to participate. Two weeks prior to data collection, the research team presented an overview of the study to teachers of selected classrooms. Following standard procedures for research at Mozambican public schools, teachers were given copies of consent and assent forms. Teachers then instructed students to take the form home to review with their caregiver and return signed if they assent to participate and their caregiver consented to their participation. Consent and assent forms contained contact information for the research team to resolve any questions before consenting or assenting to participation. The research team collected consent and assent forms from teachers prior to data collection. Of the 1320 adolescents invited to participate, 1150 (87.1%) returned consent forms. Returned consent forms were order-randomized prior to data collection. From September 9^th^ to October 2^nd^, 2019, students who had returned consent forms from two of the participating classes were gathered in an outdoor area each day, where research assistants called on them to be interviewed from the randomly ordered consent forms. We were able to evaluate a total of 493 students for eligibility. One adolescent was excluded because they returned a consent form without a caregiver signature and three were excluded because they were over 19 years old. An additional four adolecents were excluded from present analyses because they did not complete the entire interview. The final sample of 485 adolescents included in this analysis were majority female (*N* = 321, 66.2%), with a mean age of 15.9 years (SD = 1.7 years).

### Measures

We first conducted a literature review to identify screening instruments for internalizing disorders and externalizing disorders that had been previously validated in other LMIC. We then met with a team of four American and three Mozambican mental health practitioners, policy makers, and researchers specializing in disorder measurement to review the evidence considering: 1) demonstrated performance in high-income countries; 2) demonstrated performance in LMIC, with special attention to those validated in Brazil, which is, like Mozambique, a lusophone LMIC; 3) number of items, with fewer items being preferable in low-resource settings; and 4) cost per use, as LMIC are very unlikely to have funds to pay for screen administration. From these meetings, the PHQ-A, GAD-7, and SDQ were selected for assessment in the present study. The PHQ-9 is one of the most commonly used depression screening instruments [33, 34], and has been validated for use in adults in numerous LMIC, including Brazil and Mozambique [17, 24]. The PHQ-A is a nine-item version of the PHQ-9 adapted for adolescents [35] and has been demostrated to have good sensitivity and specificity for identifiying depression using adolescent self-report in high-income countries and LMIC [28, 29, 31]. The GAD-7 is a commonly used instrument to screen for anxiety in high-income countries [36, 37] that has been validated for use in LMIC adults [38, 39] and adolescents from high-income countries [30, 32] and LMIC [27]. The SDQ is a widely-used, 25-item instrument for youth (ages 4–17 years) emotional and behavioral functioning [40]. The SDQ includes five subscales which can be grouped into two 10-item subscales: an internalizing subscale (i.e. emotional and peer problems subscales) and an externalizing subscale (i.e. conduct and hyperactivity/inattention subscales) [41].The fifth subscale of the instrument, prosocial, is not included in either the internalizing nor externalizing subscales. While the SDQ includes both internalizing and externalizing subscales, it is not designed to distinguish between disorders within these diagnostic categories (i.e. depression or anxiety). The SDQ is the only open-access measure of externalizing disorders we identified and has been used in over 50 studies in Africa [42]; however, the performance of the SDQ has mixed results in both high-income country and LMIC settings [43–48]. All three instrumentsare available for use without charge.

To adapt these instruments, we followed World Health Organization procedures for translation and adaptation of psychiatric assessment instruments [49]. The adaptation team included five Mozambican psychologists (SA, CB, EC, MEF, TR) and one American global mental health researcher (KLL). We began with existing Portuguese versions from Brazil or Portugal and for all instruments, except the SDQ, made minor adjustments (e.g. verb tense, local slang for substances) to Mozambican Portuguese. All adaptations were collaboratively reviewed by a bilingual group of 9 Mozambican and 2 American mental health professionals (psychiatrists and psychologists), adjusted based on feedback, back-translated by a bilingual native English speaker not associated with the present study, and verified for accuracy to initial English versions of instruments. The translation team then conducted cognitive interviews with adolescents attending primary care clinics to assess the accuracy, appropriateness, and comprehensibility of measures [50]. We conducted cognitive interviewing using five focus groups [51] of Mozambican adolescents (*n* = 48) in which adolescents were asked a series of guide questions, followed by further probing, with regard to each item in the screening instruments: 1) Did you understand what this question was asking? 2) Was it difficult to respond to the question? 3) What do you think this question was asking? Can you repeat the question in your own words? 4) Did you understand the response options given?; 5) Were you able to find a response option you would select? 6) Were there any words in the question or response options that you didn’t understand? 7) Were there any words or expressions in the question that you found inappropriate or offensive? The translation team iteratively updated instruments based on feedback between each focus group, such that the fifth focus group reviewed and approved the finalized instruments.

We used the Portuguese version [52] of the MINI International Neuropsychiatric Interview for Children and Adolescents (MINI-KID), a structured diagnostic interview for DSM-IV and ICD-10 disorders [53], as our gold standard mental health assessment tool. We assessed presence of a current disorder on the following modules: major depressive episode (past 2 weeks), generalized anxiety disorder (past 6 months), separation anxiety disorder (past month), social phobia (past month), agoraphobia (past 6 months), panic disorder (past month), ADHD (past 6 months), conduct disorder (past 12 months), oppositional defiant disorder (past 6 months), PTSD (past month), alcohol use disorder (past 12 months), substance use disorder (past 12 months), suicidality (past month). In the present analyses, we focus only on results from modules corresponding to internalizing disorders (major depressive episode, generalized anxiety disorder, separation anxiety disorder, social phobia, agoraphobia, and panic disorder) and externalizing disorders (ADHD, conduct disorder, and oppositional defiant disorder). We also collected sociodemographic data including grade, age, gender, preferred language, religion, and household goods.

### Data collection

Trained interviewers (13 local psychologists) administered the self-report sociodemographic questionnaire, MINI-KID, and screening instruments. All participants responded to the sociodemographics questionnaire first, and were randomized to respond to either the screening battery or the MINI-KID next. Immediately following completion, a different interviewer administered the remaining measure. We invited a random selection of 49 (10%) of participants to return one-week later and be re-tested with the screening battery. All data was collected on tablets using the REDCAP electronic data collection platform [54].

Following completion of interviews, on-site research team members (SA, CB, EC, MEF, TR) provided referrals to mental health services in the neighboring primary care clinic (< 1 block from the schools) to all adolescents screened positive for any disorder on the MINI-KID. For adolescents with acute suicide risk, research team members immediately contacted caregivers and accompanied the adolescent to the psychologist at the neighboring primary care clinic.

### Data analyses

Analyses were conducted in Stata IC 14. Summary statistics of participant demographics were calculated for the total sample and for those diagnosed with depression, anxiety, and externalizing disorders (based on MINI-KID). Cronbach’s alpha statistics were calculated for each instrument as well as the SDQ internalizing and externalizing subscales to evaluate internal consistency. Individual item correlations were also evaluated, with those whose exclusion would improve the Cronbach’s alpha of the scale by > 0.01 considered for removal. Intraclass correlation coefficients (ICC) and 95% confidence intervals (CI) were calculated to assess test–retest reliability. For the purpose of assessing the criterion validity of the PHQ-A, we compared scores to diagnosis of major depresive disorder in the MINI-KID; for the GAD-7, we compared scores to diagnoses of generalized anxiety disorder, agoraphobia, social phobia, seperation anxiety, and/or panic disorder; SDQ internalizing subscale scores were compared to diagnoses of major depressive disorder, generalized anxiety disorder, agoraphobia, social phobia, seperation anxiety, and/or panic disorder; SDQ externalizing subscale scores were compared to diagnoses of conduct disorder and oppositional defiant disorder (disruptive behavior disorders) and ADHD. We generated receiver operating characteric curves (ROC) for each instrument and calculated the area under the curve (AUC). Youden’s index (J) was calculated to identify the optimal cutoff score that maximizes sensitivity and specificity for each scale [55]. Positive predictive value and negative predictive value for instruments were calculated using the prevalence in the study sample as well as a range of prevalences (1%, 5%, and 10%) identified in previous youth samples around the globe [22, 56].

## Results

### Instrument adaptation

Overall, minimal changes in content were required following cognitive interviews. Specifically, the grammatical phrasing had to be changed to “you” statements (e.g. “You felt..” instead of “Feeling…” on the PHQ-A and GAD-7) for adolescents to better understand the response options on the questionnaires. Other small changes included contextually relevant examples of possessions (e.g. pens instead of CDs).

### Participant characteristics

Of the 485 included adolescents, 441 (90.9%) reported Portuguese as their preferred language and 423 (87.8%) reported being Christian or Catholic. In total, the interviews lasted on average 17.4 min (SD = 7.2) for the screening battery and 12.6 min (SD = 7.0) for the MINI-KID. Table 1 presents the rate of disorders identified in participants through MINI-KID interviews. Internalizing disorders were over twice as common as externalizing disorders in the present sample.

**Table 1:** Participant psychiatric disorder diagnoses based on MINI-KID interviews

	Total N (%)
Internalizing Disoders	95 (19.6)
Major Depressive Episode	41 (8.5)
Anxiety^a^	85 (17.5)
Externalizing Disorders	36 (7.4)
ADHD	16 (3.3)
Disruptive Behavior Disorders^b^	32 (6.6)

^a^Includes diagnoses of generalized anxiety disorder, seperation anxiety disorder, social phobia, agoraphobia; and panic disorder

^b^includes diagnoses of conduct and oppositional defiant disorders

### Internal consistency and test–retest reliability

The PHQ-A, GAD-7, and SDQ (25 items) showed good internal consistency (Cronbach’s alpha ≥ 0.80; Table 2); whereas internal consistency for subscales was worse: moderate for the SDQ internalizing disorders (Cronbach’s alpha = 0.73) and poor for the SDQ externalizing disorders (Cronbach’s alpha = 0.65). Individual item covariance ranged from 0.19–0.20 on the PHQ-A, 0.29–0.31 on the GAD-7, 0.10–0.14 on the SDQ internalizing disorders subscale, and 0.06–0.09 on the SDQ externalizing disorders subscale (Table 3). Dropping any one item of the PHQ-A or GAD-7 did not improve internal consistency of the scale (alpha without item, Table 3). SDQ items 11 (“I have one good friend or more”), 16 (“I am nervous in new situations. I easily lose confidence”), and 23 (“I get along better with adults than with people my own age”) had low correlations with the internalizing disorders subscale (item test and item rest correlation, Table 3), and removal of each individually increased the Cronbach’s alpha of the subscale from 0.73 to 0.75 (alpha without item, Table 3). Item 7 of the SDQ (“I usually do as I am told”) had poor correlation with the externalizing disorders subscale, and its removal increased the Cronbach’s alpha of the subscale from 0.65 to 0.66.

**Table 2:** Internal Consistency and Test–retest Reliability of the PHQ-A, GAD-7, and SDQ Screening Tools in Mozambican Adolescents

Questionnaire	Internal Consistency^a^	Test–Retest Reliability^b^
PHQ-A	0.80	0.74 (0.52–0.87)
GAD-7	0.84	0.70 (0.52–0.82)
SDQ Total	0.80	0.77 (0.63–0.87)
SDQ Internalizing	0.73	0.63 (0.43–0.77)
SDQ Externalizing	0.65	0.74 (0.58–0.84)

^a^Cronbach’s alpha calculated with full sample of 485 adolescents

^b^Intraclass correlation coefficent calculated from randomly selected sub-sample of 49 adolescents tested one week after initial test

**Table 3:** Individual items correlations of the PHQ, GAD, and SDQ

Item	Item test correlation	Item rest correlation	Interitem covariance	Alpha without item
PHQ				
PHQ1	0.69	0.58	0.19	0.77
PHQ2	0.55	0.42	0.21	0.79
PHQ3	0.62	0.46	0.19	0.79
PHQ4	0.60	0.46	0.20	0.79
PHQ5	0.65	0.52	0.20	0.78
PHQ6	0.66	0.55	0.19	0.77
PHQ7	0.66	0.52	0.19	0.78
PHQ8	0.58	0.46	0.20	0.79
GAD				
GAD1	0.72	0.61	0.30	0.81
GAD2	0.69	0.56	0.31	0.82
GAD3	0.72	0.57	0.29	0.82
GAD4	0.69	0.56	0.30	0.82
GAD5	0.74	0.63	0.30	0.81
GAD6	0.71	0.60	0.30	0.81
GAD7	0.72	0.59	0.30	0.81
SDQ Internalizing				
SDQ3	0.58	0.44	0.11	0.71
SDQ6	0.59	0.45	0.11	0.70
SDQ8	0.68	0.54	0.10	0.69
**SDQ11**	**0.29**	**0.13**	**0.13**	**0.75**
SDQ14	0.74	0.64	0.10	0.68
**SDQ16**	**0.23**	**0.08**	**0.14**	**0.75**
SDQ18	0.64	0.50	0.11	0.70
SDQ19	0.51	0.40	0.12	0.71
**SDQ23**	**0.39**	**0.19**	**0.13**	**0.75**
SDQ24	0.70	0.57	0.10	0.68
SDQ Externalizing				
SDQ2	0.52	0.31	0.07	0.63
SDQ5	0.61	0.45	0.07	0.60
**SDQ7**	**0.29**	**0.13**	**0.09**	**0.66**
SDQ10	0.58	0.38	0.07	0.61
SDQ12	0.43	0.31	0.08	0.63
SDQ15	0.71	0.56	0.06	0.57
SDQ18	0.64	0.48	0.06	0.59
SDQ21	0.36	0.17	0.08	0.65
SDQ22	0.41	0.27	0.08	0.64
SDQ25	0.30	0.10	0.09	0.67

Internalizing, internalizing disorders; Externalizing, externalizing disorders

The correlation between test and retest scores (Table 2) was moderate for the PHQ-A, GAD-7, full SDQ, and SDQ externalizing subscale (ICC ≥ 0.70) but poor for the SDQ internalizing subscale (ICC = 0.63, 95%CI = 0.43–0.77).

### Criterion validity

A summary of the performance of the PHQ-A, GAD-7, and SDQ as compared to MINI disorder diagnoses is shown in Table 4. The area under the ROC curve (AUC) for all scales was > 0.70 (full ROC curves shown in Additional Fig. 1). Youdin’s index identified an optimal cutoff score of 8 for the PHQ-A, 5 for the GAD-7, 10 for the SDQ internalizing and 9 for the SDQ externalizing subscales. At these cutoff scores, sensitivity and specificity of the PHQ-A for depression, GAD-7 for anxiety, SDQ internalizing subscale for internalizing disorders, and SDQ externalizing subscale for externalizing disorders were all acceptable (> 0.70). The SDQ internalizing subscale showed slightly higher sensitivity but lower specificity for adolescents with depression (sens. = 0.83, spec. = 0.68) than adolescents with anxiety (sens. = 0.74, spec. = 0.72); the SDQ externalizing subscale had slighty lower specificity but higher sensitivity for adolescents with disruptive behavior disorders (sens. = 0.72, spec. = 0.70) than ADHD (sens. = 0.75, spec. = 0.57). Positive predictive values (PPV) and negative predictive values (NPV) for the scales at the identified optimal cutoff scores for different disorder prevalences can be found in Additional Table 1. Details on the performance of scales at all possible cutoff scores are in Additional Table 2.

**Table 4:** Performance of the PHQ-A, GAD-7, and SDQ Screening Tools in Mozambican Adolescents

	AUC (95% CI)	Cutoff^a^	Sensitivity	Specificity	% Correctly Classified
PHQ-A	0.85 (0.76–0.90)	8	0.78	0.80	80.2
GAD-7	0.84 (0.79–0.89)	5	0.81	0.72	73.6
SDQ Internalizing Subscale					
Any Internalizing Disorder^b^	0.80 (0.75–0.85)	10	0.76	0.74	74.3
Depression	0.81 (0.74–0.87)	10	0.83	0.68	69.7
Anxiety	0.78 (0.73–0.84)	10	0.74	0.72	72.6
SDQ Externalizing Subscale					
Any Externalizing Disorder^c^	0.72 (0.65–0.80)	9	0.78	0.59	60.4
ADHD	0.70 (0.60–0.81)	9	0.75	0.57	57.9
Disruptive Behavior Disorders	0.74 (0.67–0.82)	10	0.72	0.70	70.1

*Abbreviations*: *AUC* Area under the curve, *CI* Confidence interval, *PHQ-A* Patient Health Questionnaire for Adolescents, *GAD-7* Generalized Anxiety Disorders 7, *SDQ* Strengths and Difficulties Questionnaire

^a^Optimal cutoff score determined by Youdin’s index

^b^Diagnoses of depression and/or anxiety

^c^Diagnoses of conduct disorder, oppositional defiant disorder, and/or ADHD

## Discussion

We sought to evaluate screening measures for internalizing and externalizing disorders that could be used easily and effectively by non-specialists in Mozambique, i.e., open access, few items, established validity in international settings. We selected the PHQ-A and GAD-7 owing to their strong performance identifying depression and anxiety symptoms, respectively, in adolescents of high-income countries and LMIC [27–32]. As in previous studies, the PHQ-A and GAD-7 in our sample showed acceptable internal consistency and test–retest reliability. Moreover, both instruments demonstrated good sensitivity and specificity for depression and anxiety, respectively.

Additionally, we selected the SDQ because it has demonstrated satisfactory performance and is one of the most commonly used instruments globally for assessing both internalizing disorders and externalizing disorders [48], and to our knowledge the only brief screen for externalizing disorders without a cost-per-use.In African youth populations, the internal consistency of the SDQ has been demonstrated to vary widely (Cronbach’s alpha = 0.18–0.89) [42]. In our sample, internal consistency of the full SDQ (Cronbach’s alpha = 0.80) and internalizing subscale (Cronbach’s alpha = 0.73) were good, while that of the externalizing subscale was weaker (Cronbach’s alpha = 0.65). These findings are similar to data from self-report, non-clinical adolescent samples in high-income countries and other LMIC, in which acceptable internal consistency of the full instrument but not the subscales has been found [57–63]. Moreover, previous research has demonstrated that the factor structure of the SDQ differs cross-culturally, suggesting that individual SDQ items may be more culturally-dependent than others and that further testing of the measure in diverse populations is required [64]. Our assessment of the performance of individual items of the SDQ subscales identified four questions (items 7, 11, 16, and 23) that had poor correlation with other items. However, removal of the items individually only minimally improved internal consistency of the subscales (Cronbach’s alpha = 0.73 to 0.75 for internalizing subscale and Cronbach’s alpha = 0.65 to 0.66 for externalizing subscale). Studies of adolescents from diverse cultural settings, including South Africa, Norway, Israel, Germany, and China, similarly found that item 7 (“I usually do as I am told”) and items 11 and 23 (“I have one good friend or more” and “I get along better with adults than with people my own age”) had poor factor loadings for conduct problems and peer relationships, respectively [47, 57, 58, 61, 65, 66], suggesting these items may be generally less predictive of problem areas. Previous research in Zambian youth described challenges in determining a local linguistic equivalent to translate item 16 (“I am nervous in new situations. I easily lose confidence”) [67]. Because our cognitive interviews with adolescents did not reveal any difficulties in interpretation of this item, we do not believe its inconsistency is due to a translation issue. However, further research is needed to determine if this item is not culturally relevant or if its interpretation is disparate among Mozambican youth.

Finally, the optimal cutoff score for each instrument in our sample was lower than recommended cutoff scores from high-income countries [30, 31, 68], a finding that has been demonstrated in multiple LMIC for both children [29] and adults [24, 69, 70]. It is important to note, though, that while the optimal cutoff scores in the present study provide the best balance of sensitivity and specificity for identifying a disorder in this population, they do not account for severity of symptoms or functioning. As such, these optimal cutoff scores are more appropriate for certain screening purposes, e.g., estimating disorder prevalence in the general adolescent population, than others, e.g. identifying adolescents who should receive treatment in a resource-limited setting where inclusion of adolescents with mild symptoms could overwhelm the system. Still, we recommend proceeding with caution when considering increasing the cutoff score, owing to the fairly steep decline in sensitivity using higher cutoff scores. For example, at the recommended cutoff score of 10 for moderate depression, the sensitivity of the PHQ-A in our sample drops to 59%; at the recommended cutoff score of 10 for moderate anxiety, the sensitivity of the GAD-7 in our sample drops to 46%. Therefore, screening purpose, expected disorder prevalence, and available treatment resources should be carefully considered before selecting a particular cutoff score for instrument implementation.

Our study had a few important limitations. First, adolescents were recruited at public secondary schools in urban and peri-urban areas, and thus may not be generalizable to Mozambican adolescents who do not attend school or who live in rural regions of the country. The cognitive interviews conducted to establish comprehensibilty of the questions was done with adolescents who were attending primary care clinics in Maputo City, who represent both adolescents in and out of school. Still, though the instruments may be understood by these adolescents, this does not indicate they would perform similarly in indentifying mental disorders in this population. Additionaly, all instruments were validated in Portuguese, which was the predominant language of the study sample but is not the primary language of many Mozambicans. We therefore recommend that future studies aim to assess performance of these instruments in rural, non-school attending, and non-Portuguese speaking Mozambican adolescents. Finally, the timeframe of symptom assessment in the screening instruments differed from the MINI-KID for some disorders (e.g. MINI-KID conduct disorder = past 12 months, SDQ externalizing disorders = past 6 months). Still, our data demonstrated acceptable to good criterion validity of all screening instruments.

Despite these limitations, the present study had a number of strengths. For one, this is the first study to validate any type of mental health screening instrument in Mozambican adolescents. Moreover, our instrument adaptation was driven by input from local mental health experts and adolescents. We also used a gold standard (the MINI-KID) to establish criterion validtity. Finally, we included a random selection of adolescents from schools that spanned a wide range of adolescents ages (12–19).

The PHQ-A, GAD-7, and full SDQ demonstrated good internal and test–retest reliability. All scales showed adequate to good criterion validity based on diagnoses made using the MINI-KID interview. These findings indicate these instruments can serve as a useful tool for rapidly assessing adolescent mental health problems in Mozambique without the need of specialized providers and may be of interest to other LMIC looking to expand task-shifted mental health services for adolescents. In terms of selecting when to use the PHQ-A, GAD-7 or SDQ, we recommend that the specific needs of the population being assessed be used to inform decision-making. For example, in a setting where it is important to determine whether a child has depression and/or anxiety, such as when a provider is selecting their specific treatment approach, we recommend the use of the PHQ-A and GAD-7. However, if the need is to identify youth with general internalizing and/or externalizing problems, such as in a community-based screen to provide referrals to mental health services, it may be preferable to use the SDQ.

## Conclusions

Here, we for the first time adapt and evaluate the performance of mental health screening measures in Mozambican adolescents and add to the very limited literature on screening measures in LMIC youth, who account for the vast majority of the world’s young people. Our results suggest that the PHQ-A, GAD-7, and SDQ have satisfactory psychometric properties for screening for symptoms of depressive anxiety, internalizing, and externalizing disorders, respecitvely. Future research should focus on the performance of these measures in rural and non-Portuguese-speaking Mozambican adolescents, as well as their performance in other LMIC.

## Supplementary Information

**Additional file 1.**

## Data Availability

The dataset generated during the current study are not publicly available due to Mozambican ethics committee regulations, but are available from the corresponding author (Kathryn Lovero, kll2153@cumc.columbia.edu) on reasonable request.
