# Supplementary material for: Validation of brief screening instruments for internalizing and externalizing disorders in Mozambican adolescents
Source: BMC Psychiatry. 2022 Aug 12;22:549. doi: 10.1186/s12888-022-04189-3 (PMC9373392; doi:10.1186/s12888-022-04189-3)
Supplement: Supplementary file 1 — Additional file 1. [file 12888_2022_4189_MOESM1_ESM.docx]

**Additional Tables and Figure**

Additional Table 1. Positive predictive values and negative predictive values of the PHQ-A, GAD-7, and SDQ using the sample population prevalance and a range of other prevalences.

|  |  | Sample Prevalence^b^ | | 1% Prevalence | | 5% Prevalence | | 10% Prevalence | |
| --- | --- | --- | --- | --- | --- | --- | --- | --- | --- |
|  | Cutoff^a^ | PPV | NPV | PPV | NPV | PPV | NPV | PPV | NPV |
| PHQ-A | 8 | 0.27 | 0.98 | 0.04 | 1.0 | 0.17 | 0.99 | 0.31 | 0.97 |
| GAD-7 | 5 | 0.38 | 0.95 | 0.03 | 1.0 | 0.13 | 0.99 | 0.24 | 0.97 |
| SDQ |  |  |  |  |  |  |  |  |  |
| Internalizing^c^ | 10 | 0.41 | 0.93 | 0.03 | 1.0 | 0.13 | 0.98 | 0.24 | 0.97 |
| Externalizing^d^ | 9 | 0.13 | 0.97 | 0.02 | 1.0 | 0.9 | 0.98 | 0.17 | 0.96 |

Abbreviations: PHQ-A, Patient Health Questionnaire for Adolescents; GAD-7 Generalized Anxiety Disorders 7; SDQ, Strengths and Difficulties Questionnaire. ^a^Optimal cutoff score determined by Youdin’s index; ^b^Sample prevalence of depression = 8.5, anxiety = 17.5, internalizing disorders = 19.6, externalizing disorders = 7.4; ^c^Diagnoses of depression and/or anxiety; ^d^Diagnoses of conduct disorder, oppositional defiant disorder, and/or ADHD

Additional Table 2. Instrument performance at all possible cutoff scores.

| Cutoff | Sensitivity | Specificity | % Correctly Classified |
| --- | --- | --- | --- |
| PHQ-A |  |  |  |
| ≥0 | 1.00 | 0.00 | 8.5 |
| ≥1 | 1.00 | 0.13 | 20.0 |
| ≥2 | 1.00 | 0.25 | 31.2 |
| ≥3 | 1.00 | 0.40 | 45.5 |
| ≥4 | 0.93 | 0.50 | 53.7 |
| ≥5 | 0.88 | 0.61 | 63.0 |
| ≥6 | 0.85 | 0.70 | 70.9 |
| ≥7 | 0.80 | 0.76 | 76.5 |
| ≥8 | 0.78 | 0.80 | 80.2 |
| ≥9 | 0.61 | 0.84 | 82.4 |
| ≥10 | 0.59 | 0.89 | 86.2 |
| ≥11 | 0.46 | 0.92 | 88.2 |
| ≥12 | 0.32 | 0.95 | 89.5 |
| ≥13 | 0.27 | 0.96 | 90.1 |
| ≥14 | 0.22 | 0.97 | 90.5 |
| ≥15 | 0.20 | 0.97 | 90.7 |
| ≥16 | 0.12 | 0.98 | 90.9 |
| ≥17 | 0.10 | 0.98 | 90.9 |
| ≥18 | 0.05 | 0.98 | 90.5 |
| ≥19 | 0.02 | 0.99 | 90.5 |
| ≥20 | 0.02 | 0.99 | 90.7 |
| ≥22 | 0.00 | 0.99 | 90.5 |
| ≥24 | 0.00 | 0.99 | 90.9 |
| ≥27 | 0.00 | 1.00 | 91.3 |
| >27 | 0.00 | 1.00 | 91.5 |
| GAD-7 |  |  |  |
| ≥0 | 1.00 | 0.00 | 17.53 |
| ≥1 | 0.96 | 0.20 | 32.99 |
| ≥2 | 0.94 | 0.36 | 46.19 |
| ≥3 | 0.93 | 0.49 | 56.70 |
| ≥4 | 0.88 | 0.63 | 67.22 |
| ≥5 | 0.81 | 0.72 | 73.61 |
| ≥6 | 0.73 | 0.79 | 77.94 |
| ≥7 | 0.68 | 0.84 | 80.82 |
| ≥8 | 0.64 | 0.88 | 83.71 |
| ≥9 | 0.59 | 0.92 | 85.98 |
| ≥10 | 0.46 | 0.94 | 85.15 |
| ≥11 | 0.34 | 0.96 | 84.74 |
| ≥12 | 0.24 | 0.98 | 84.54 |
| ≥13 | 0.18 | 0.98 | 83.51 |
| ≥14 | 0.16 | 0.99 | 84.12 |
| ≥15 | 0.13 | 1.00 | 84.33 |
| ≥16 | 0.08 | 1.00 | 83.51 |
| ≥17 | 0.07 | 1.00 | 83.30 |
| ≥18 | 0.06 | 1.00 | 83.09 |
| ≥19 | 0.04 | 1.00 | 82.68 |
| ≥20 | 0.02 | 1.00 | 82.68 |
| ≥21 | 0.01 | 1.00 | 82.68 |
| >21 | 0.00 | 1.00 | 82.47 |
| SDQ - Internalizing Disorders | |  |  |
| ≥1 | 1.00 | 0.00 | 19.59 |
| ≥2 | 1.00 | 0.01 | 20.41 |
| ≥3 | 1.00 | 0.03 | 21.86 |
| ≥4 | 1.00 | 0.06 | 24.54 |
| ≥5 | 0.99 | 0.17 | 32.99 |
| ≥6 | 0.94 | 0.31 | 43.51 |
| ≥7 | 0.88 | 0.44 | 52.37 |
| ≥8 | 0.85 | 0.57 | 62.27 |
| ≥9 | 0.79 | 0.67 | 69.48 |
| ≥10 | 0.76 | 0.74 | 74.23 |
| ≥11 | 0.66 | 0.80 | 77.53 |
| ≥12 | 0.59 | 0.86 | 80.41 |
| ≥13 | 0.45 | 0.90 | 81.03 |
| ≥14 | 0.35 | 0.93 | 81.86 |
| ≥15 | 0.25 | 0.95 | 81.24 |
| ≥16 | 0.14 | 0.98 | 81.24 |
| ≥17 | 0.09 | 0.99 | 81.44 |
| ≥18 | 0.04 | 1.00 | 81.24 |
| SDQ - Externalizing Disorders | |  |  |
| ≥1 | 1.00 | 0.00 | 7.42 |
| ≥2 | 1.00 | 0.00 | 7.63 |
| ≥3 | 1.00 | 0.02 | 9.07 |
| ≥4 | 1.00 | 0.03 | 10.10 |
| ≥5 | 1.00 | 0.10 | 16.49 |
| ≥6 | 0.97 | 0.18 | 24.33 |
| ≥7 | 0.92 | 0.36 | 40.00 |
| ≥8 | 0.83 | 0.45 | 48.25 |
| ≥9 | 0.78 | 0.59 | 60.41 |
| ≥10 | 0.67 | 0.70 | 69.69 |
| ≥11 | 0.56 | 0.78 | 76.29 |
| ≥12 | 0.39 | 0.84 | 80.21 |
| ≥13 | 0.22 | 0.89 | 83.71 |
| ≥14 | 0.19 | 0.92 | 87.01 |
| ≥15 | 0.08 | 0.95 | 88.87 |
| ≥16 | 0.06 | 0.98 | 91.34 |
| ≥17 | 0.00 | 0.99 | 91.96 |

Abbreviations: PHQ-A, Patient Health Questionnaire for Adolescents; GAD-7 Generalized Anxiety Disorders 7; SDQ, Strengths and Difficulties Questionnaire.

Additional Figure 1. Receiver Operating Charactertistic curves for detection of depression by the PHQ-A (A), anxiety disorders by the GAD-7 (B), and internalizing disorders (C) and externalizing disorders (D) by the corresponding SDQ subscales among Mozambican adolescents.

A. B.

C. D.

Abbreviations: PHQ-A, Patient Health Questionnaire for Adolescents; GAD-7 Generalized Anxiety Disorders 7; SDQ, Strengths and Difficulties Questionnaire.
